# Measuring Device and Material ZT in a Thin-Film Si-Based Thermoelectric Microgenerator

**DOI:** 10.3390/nano9040653

**Published:** 2019-04-24

**Authors:** Pablo Ferrando-Villalba, Antonio Pablo Pérez-Marín, Llibertat Abad, Gustavo Gonçalves Dalkiranis, Aitor F. Lopeandia, Gemma Garcia, Javier Rodriguez-Viejo

**Affiliations:** 1 Departament de Física, Universitat Autònoma de Barcelona, 08193 Cerdanyola del Vallès, Spain; pablo.ferrandovillalba@imec.be (P.F.-V.); apperezmarin@gmail.com (A.P.P.-M.); dalkiranis@gmail.com (G.G.D.); gemma.garcia@uab.cat (G.G.); Javier.rodriguez@uab.cat (J.R.-V.); 2 Institut de Microelectrònica de Barcelona-Centro Nacional de Microelectrónica, CSIC, 08193 Cerdanyola del Vallès, Spain; llibertat.abad@imb-cnm.csic.es

**Keywords:** thermoelectric characterization, thermoelectric generator, Si thin films

## Abstract

Thermoelectricity (TE) is proving to be a promising way to harvest energy for small applications and to produce a new range of thermal sensors. Recently, several thermoelectric generators (TEGs) based on nanomaterials have been developed, outperforming the efficiencies of many previous bulk generators. Here, we presented the thermoelectric characterization at different temperatures (from 50 to 350 K) of the Si thin-film based on Phosphorous (n) and Boron (p) doped thermocouples that conform to a planar micro TEG. The thermocouples were defined through selective doping by ion implantation, using boron and phosphorous, on a 100 nm thin Si film. The thermal conductivity, the Seebeck coefficient, and the electrical resistivity of each Si thermocouple was experimentally determined using the in-built heater/sensor probes and the resulting values were refined with the aid of finite element modeling (FEM). The results showed a thermoelectric figure of merit for the Si thin films of zT = 0.0093, at room temperature, which was about 12% higher than the bulk Si. In addition, we tested the thermoelectric performance of the TEG by measuring its own figure of merit, yielding a result of *ZT* = 0.0046 at room temperature.

## 1. Introduction

Thermoelectricity is the ability of some materials to produce voltage when exposed to a temperature gradient, and conversely, to produce a temperature gradient when electrons pass through them. The thermoelectric figure of merit of a material establishes the efficiency in energy conversion of the material, and it is defined as $\mathrm{ZT}=\frac{{\sigma S}^{2}}{k}$, where σ is the electrical conductivity, S is the Seebeck coefficient and k is the thermal conductivity. As apparent from the formula, increasing σ and S and reducing k leads to a higher figure of merit and thus to a better conversion efficiency.

During the last two decades, the interest in thermoelectricity has exponentially grown due to the emergence of nanotechnology. Nanoscale and nanostructured materials show several non-classic transport effects, both thermal and electric, that can be exploited to improve their thermoelectric performance. The most important effect is the enhancement of phonon scattering at boundaries and interfaces, which reduces the thermal conductivity while maintaining a practically unaffected electrical conductivity [1,2]. Additionally, nanomaterials can exhibit electron confinement [3], an increased ZT via electronic band engineering [4], an enhanced Seebeck coefficient via dislocations in nanowires [5], surface states [6] and an enhanced phonon scattering via surface decoration [7], all of which favor ZT.

As a result, new thermoelectric generators (TEGs) have been designed and fabricated over the past years, benefitting from the advantages of nanomaterials. To date, various classes of thermoelectric semiconductor compounds have been developed, such as bismuth telluride-based materials for low temperature applications [8,9,10,11,12], or silicon-based materials for higher temperatures [13,14,15]. These devices can have either a vertical [16] or planar [13] geometry, meaning that the temperature gradient is either set out-of-plane or in-plane with the substrate. Generally, the in-plane geometries allow for higher temperature gradients, owing to their higher dimensions in the direction of the heat flow, and thus achieving an enhanced power output [17]. In addition, top-down fabricated TEGs show a higher yield thanks to their enhanced reproducibility compared with the bottom-up TEGs [16]. On the other hand, these TEGs can be complementary metal-oxide-semiconductor (CMOS)-compatible [13,18] if the active material complies with the technology standards, thus allowing for the integration of generators with low-power consuming devices, such as wearables or body sensors, as well as the integration of microcoolers with microelectronic devices, such as lasers [19].

Several techniques have been developed to evaluate the performance of a TEG [20,21], measuring the efficiency and the figure of merit (ZT), while avoiding some of the side effects, such as the Thomson effect. However, an alternative means of characterization consists of separately measuring the three extrinsic parameters related ZT: the total heat conductance from the hot side to the cold side, the electrical resistance (produced by the active material plus the contact resistances), and the total Seebeck coefficient produced by all of the TEG legs. This is an advantageous approach when treating with microTEGs, where thermal dynamics are much faster than in bulk systems, the heat fluxes can be better controlled, and the thermal contact resistance between deposited films can be neglected.

In this paper, we characterized the performance of a previously reported planar and CMOS-compatible TEG [18], and measured the figure of merit of the active material, which consisted of p- and n-doped 100 nm thin-film Si. The comparison between the ZT of the complete TEG with the zT of the active nanoSi, allowed us to assess the grade of optimization, as well as the amount of room left for increasing the performance of the device. Taking measurements of the three individual parameters composing the figure of merit was made possible by taking advantage of an in-built heating grid that created a known ΔT between the central part (hot side) and the frame (cold side). Experimental measurements were combined with a detailed 3D finite element model (FEM) to extract the contributions of structural materials in the thermal conduction and to calculate the real temperature difference between the Si ends, thus correcting the resulting Seebeck and thermal conductance measurements. A 3D FEM was also developed to subtract the current crowding effect in the electrical conductance measurements.

## 2. Experimental Methods

**Figure 1:**
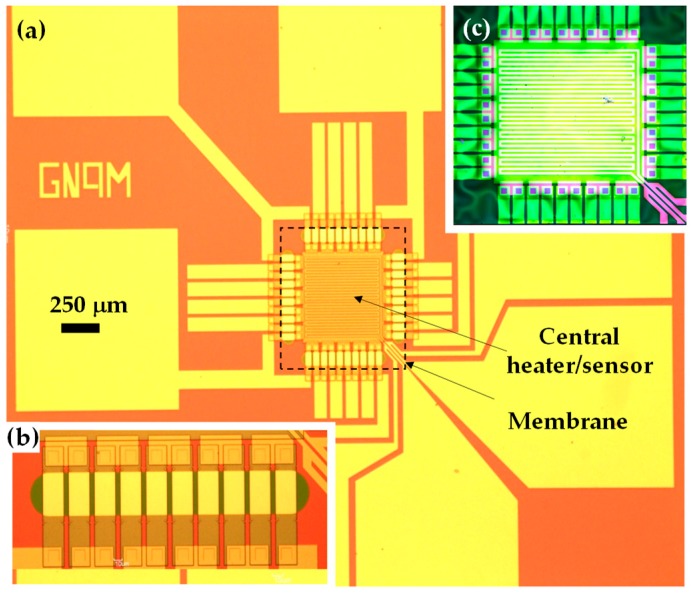
(**a**) Micrograph of the front of the thermoelectric generator (TEG) being tested, indicating the membrane area and the central heater/sensor Au grid locations. (**b**) Detail of the thin film strips that conform to the 5 n-p thermocouples that were found in each side of the squared membrane. (**c**) Image of the free-standing membrane take from the back.

The characterized device was a planar thin-film TEG, where the active materials are p- and n-doped Si strips, with doping concentrations around 2 × 10^19^ atoms/cm^2^ (Figure 1). A 400 nm thick SiO_2_ film was used as a structural material that held the Si strips and prevented them from breaking throughout the fabrication process. The contact lines were made of a 200 nm thick Au film, and the ohmic contact with the Si was favored by creating a NiSi alloy in the Si open-via, prior to the Au deposition. Since this was a test device, it also includes a grid in the center in order to create controlled temperature gradients in the Si strips. The complete fabrication of this device is explained elsewhere [18].

The measurement of the electrical resistivity was performed by feeding the Si thermocouples with a current of 1 μA and measuring the voltage drop using the offset compensation mode of the source (Keithley 2400, Keithley, Cleveland, OH, USA). In order to extract the resistivity of the material, we created a 3D finite element model (FEM) with COMSOL (Version 4.2, COMSOL AB, Stockholm, Sweden), which accounted for the current crowding effect at the contacts. The following methodology was used. First, the Au resistivity was set to the resistivity being measured in the grid, and the contact resistivity between Au/Si was set from a previous measurement. Next, the electrical resistivity of p-Si and n-Si was set equal ($\rho_{p-\mathrm{Si}}=\rho_{n-\mathrm{Si}}=\rho_{\mathrm{Si}}$). This resistivity was scanned and the values at which the resistance of the model resembled the resistivities being measured were selected as the real resistivity values.

The thermal conductivity was measured by heating up the central grid (by feeding with current) and measuring the voltage in 4-wires using a Keithley 2425. After the temperature calibration of the grid as a resistance temperature detector (RTD) was performed, the temperature was measured from the change in resistance at each point. The thermal conductance was then calculated as G=P/ΔT, and the thermal conductivity was found by applying the geometric factor of the 40 Si strips. However, this experiment alone lacks accuracy since the measured temperature difference was not exactly the one between the Si ends due to the temperature inhomogeneity in the grid, and because not all of the heat flux was evacuated through the Si strips, but partly through the SiO_2_ and the Au instead. In order to correct these effects, a second 3D FEM was created using COMSOL. After assigning plausible thermal conductance values to all of the materials for the different temperatures (see Table 1), the thermal conductivity value of the doped Si was swiped until finding a concordance in thermal conductance with that of the real experimental measurements.

The Seebeck coefficient was measured by heating the central grid of the TEG and then measuring the Seebeck voltage created by the temperature difference with a Keithley 2182A. The measured value was extracted using, $S=V_{S}/n\Delta T$ where n=40 (the number of Si legs). Once again, the second 3D FEM model had to be used owing to the non-homogeneous temperature in the grid (see Appendix B).

All the measurements were carried out in a He Cryostat at high vacuum conditions (10^−5^ mbar). For each measurement, the temperature of the sample holder was stabilized with an uncertainty lower than 0.01 K.

## 3. Results and Discussion

**Figure 2:**
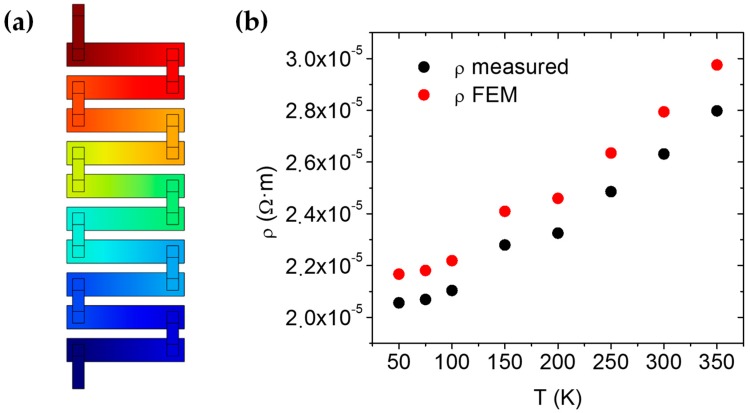
(**a**) Image of the voltage distribution in the modeled thermocouples. (**b**) Comparison between the measured resistivity (calculated using the distance between centers of the contacts as thermocouple length) and the modeled resistance.

The results of the electrical resistivity of the Si are shown in Figure 2. The measured values of ρ were calculated by multiplying the measured resistance (R) by the section of the strip (50 × 0.1 m^2^) and dividing it by the distance between the centers of the contacts (200 m). Nevertheless, the current crowding effect produced a higher density of current near the border of the contact, which reduced the distance traveled by the carriers inside the Si strip. Furthermore, the contact resistance was enhanced due to the reduction of the effective section. As these effects were considered in the simulation, the FEM-corrected ρ values were more precise than the ones directly measured. It is also worth noting that the Si behaves as a metal, since the resistance grows with the temperature, thus confirming the correct doping of the strips.

**Figure 3:**
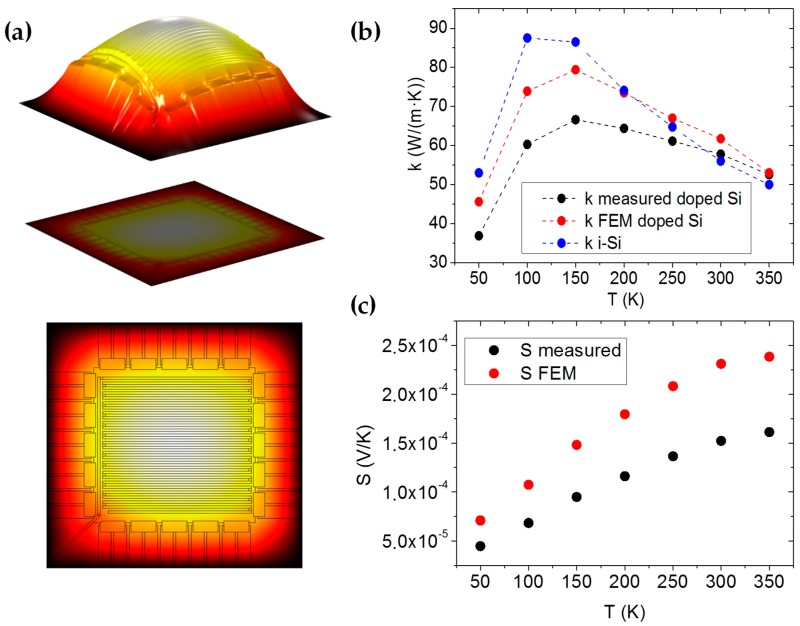
(**a**) Temperature distribution in the TEG (white is hot). (**b**) Comparison between the measured thermal conductivity k, the modeled one and an intrinsic Si film. (**c**) Comparison between the measured Seebeck coefficient k and the modeled coefficient.

The thermal conductivity was measured and then corrected using the second model developed with COMSOL (Figure 3). In Figure 3b, both results of the thermal conductivity can be seen. The two effects mentioned in the methods section have the following implications. First, the structural materials (mainly the SiO_2_) and the Au lines were responsible for a considerable part of the heat flow from the center to the substrate. Subtracting this effect, reduced the thermal conductivity of the Si. On the other hand, the inhomogeneity in the temperature of the heating grid produced a higher temperature in the center than in the limits of the grid. Thus, the measured ΔT was higher than the ΔT occurring along the silicon strips. Correcting this effect increased the Si thermal conductivity. The two effects discussed neutralized each other quite well, and the corrected thermal conductivity did not substantially differ from the raw measured value. The FEM-corrected values show the peak characteristic of crystalline materials, slightly shifted to higher temperatures compared to the intrinsic bulk Si, and the resulting values fit well with the literature data [24,25,26,27,28,29].

In the case of the Seebeck coefficient, only the effect of the temperature inhomogeneity on the grid played a role in the corrected value, and since the real ΔT along the Si strips was lower than the measured value, the S increased by a factor of around 1.5 (Figure 3c).

**Figure 4:**
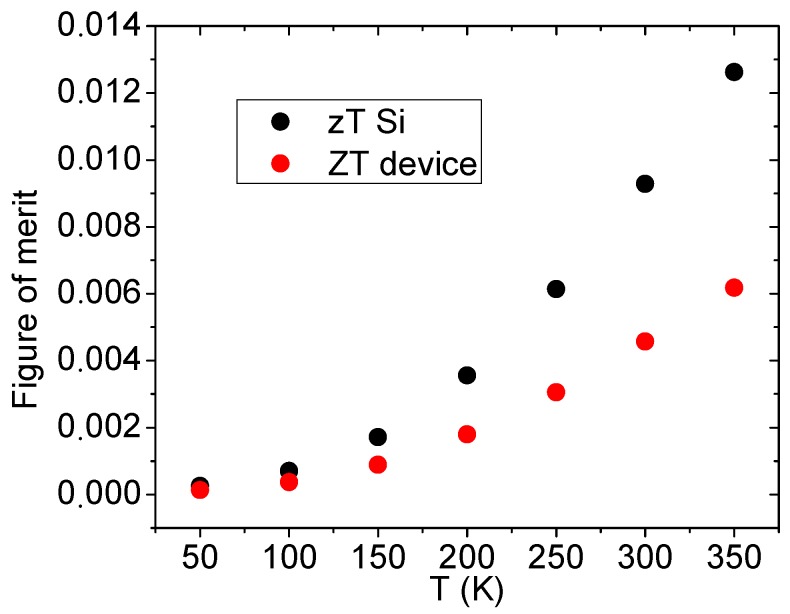
Comparison of the zT of the Si and the *ZT* of the device.

With these corrected parameters, the zT of the material was calculated (Figure 4), showing a value of 0.0093 at 300 K, which upgrades the bulk Si values [30]. Although this value was expected to be much higher due to the reduction of the thermal conductivity in thin films, the power factor $S^{2}/\rho$ was not as high as expected. Nonetheless, fabricating a device with even thinner films should increase the performance of the material. It is worth noting that this strategy will encounter technical difficulties when trying to fabricate thinner doped films, and alternative doping techniques should be considered. In addition, nucleation in extremely thin Si films due to the high temperatures used in the fabrication process should be prevented.

The figure of merit of the Si strips (zT) could now be compared to that of the whole device (ZT). This ZT is calculated as:

$$ZT=\frac{S_{Device}^{2}}{R_{Device}G_{Device}}$$

where $S_{Device}$ is the Seebeck coefficient of the whole generator, $R_{Device}$ is the measured resistance of the thermocouples (including the contact resistances) and $G_{Device}$ is the measured thermal conductance. This was the practical figure of merit of the generator, which was related to the maximum efficiency that it can perform at.

The comparison of ZT to the Si zT revealed how optimized the generator was. ZT should always be smaller than zT since a complete device performs worse than the material itself, due to side effects such as contact resistance or extra thermal conductance. In our case, the coefficient zT/ZT was always between 1.9 and 2 (in all of the temperatures measured), meaning that the efficiency of the generator was about two times lower than the optimal design. In order to improve this value, the structural thermal conductance must be suppressed by reducing the thickness and width of the supporting SiO_2_ structure, as already stated for the microcoolers’ performance [19]. However, since a final use TEG device does not require the inclusion of an Au grid, the performance will improve in any case. On the other hand, reducing the electrical contact resistance will likely slightly increase the TEG performance. The easiest way of achieving contact resistance reduction will be to enlarge the width of the contacts, as increasing the height may not produce any improvement due to the current crowding effect.

## 4. Conclusions

We characterized a micro-TEG, in which the active materials were p-Si and n-Si thin films. The performance of the active material was characterized by measuring the individual parameters of the figure of merit. Two FEM models were developed to correct the crowding effect in the electrical contacts and the temperature inhomogeneity in the grid, as well as to subtract the heat flow in the structural materials. The figure of merit of the TEG was compared to that of the active nanoSi, showing a three-fold reduction. It is noted that further optimization can be attempted by limiting heat flow lost through the structural materials.

## Figures and Tables

**Table 1:** Values of the physical properties used in all of the finite element modeling (FEM) simulations. The thermal conductivity of the gold was calculated from the measured resistivity using the Wiedemann–Franz law with L = 2.44 × 10^−8^ W/K. The contact resistivities were also measured at room temperature $\rho_{C}$ = 3.74 × 10^−9^ m^2^ (see Appendix A). The data from the references has been extrapolated.

T	$\rho_{Au}$	$k_{i-Si,100nm}$ [22]	$k_{Au}$	$k_{SiO_{2}}$ [23]
K	Ω·m	W/(m·K)	W/(m·K)	W/(m·K)
50	2.821 × 10^−8^	53	31.51	0.317
100	3.258 × 10^−8^	87.5	54.57	0.628
150	3.680 × 10^−8^	86.5	72.47	0.910
200	4.067 × 10^−8^	74.1	87.44	1.180
250	4.497 × 10^−8^	64.75	98.85	1.335
300	4.890 × 10^−8^	56	109.08	1.410
350	5.282 × 10^−8^	50	116.2	1.45

**Figure A1:**
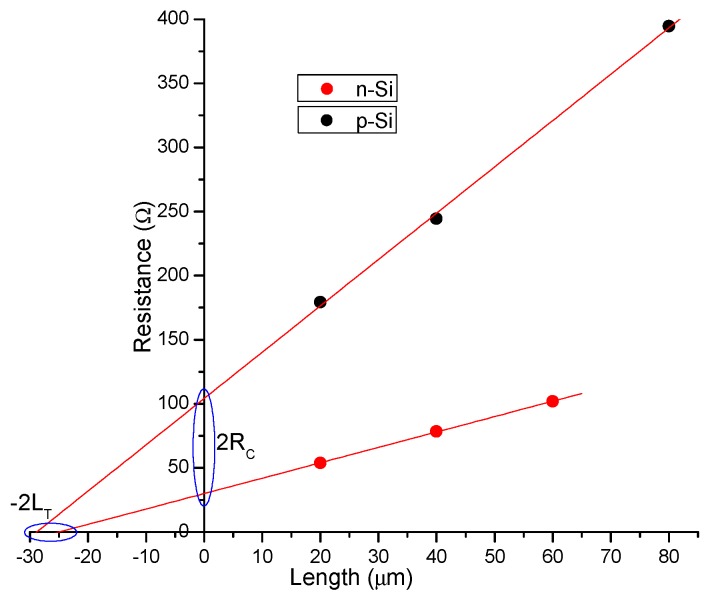
Contact resistance measurements using the transmission line method.

## Appendix A. Measurement of the Contact Resistivity

The contact resistivity was measured in n-Si and p-Si using the transmission line model. This model consisted of measuring the resistance of the material being tested by using increasingly separated metallic lines. This way, the electrical resistance of the material could be ruled out by fitting the resistance measurements with a line and achieving a value at length 0. This value is twice that of the contact resistance ($2R_{C})$. In order to calculate the electrical conductivity, a formula was derived that took into account the electron crowding effect at the borders of the electrodes. In this sense, the point at which the fitted line crossed the abscissa equals $-2L_{T}$, where $L_{T}$ (the transfer length) is the average distance that an electron travels in the semiconductor beneath the metallic lines. The formula for acquiring the contact resistivity is:

$$\rho_{C}=R_{C}L_{T}W$$

where W is the width of the contact.

The thermal contact resistance on p-Si was not measured with the actual doping level used in the TEG. After the first test, depicted in Figure A1, the implantation dose of the p-Si was increased three times in order to achieve the same doping level as in the n-Si. The contact resistance measured at room temperature was $\rho_{C}$ = 3.74 × 10^−9^ Ω·m^2^. Thus, and according to the literature, we can assume that the contact resistivity to be similar in both n- and p-Si [31] and that they have a weak temperature dependence, especially for samples with high doping levels. Since the weight of the contact resistances compared with the total Si strip resistance was smaller than 1.5%, we can assume the measured value without introducing large errors in the simulation.

## Appendix B. COMSOL Model

Two 3D models were developed using COMSOL Multiphysics.

In the first model, the physics described by the partial differential equations (PDE) was based on the module of Electrical currents, and the study was performed in steady state. The modeled system was one fourth of the Si strips circuit with the corresponding gold contacts. The contact area of 25 × 25 microns was respected. The simulation consisted of feeding the circuit with a current, and probing the voltage to calculate the effective resistance. This was done for the different temperatures by changing the material properties accordingly.

In the second model, the physics described by the PDE was based on the module of Joule Heating, and the study was performed in the steady state. For calculating the thermal conductivity of the doped Si, first the heater was fed with a current and the temperature was probed throughout it. The value of the thermal conductivity was scanned, and the correct value was selected as that which produces the same measured thermal conductance as in the real experiment.

In order to measure the real Seebeck coefficient, the simulation consisted of feeding a current to the heater using the same parameters as in the previous simulation, plus the correct value for the thermal conductivity of the doped Si. The temperature was probed both throughout the heater and between the ends of the exposed region of the Si strips. The real Seebeck coefficient was calculated as follows:

$$S_{real}=S_{measured}\cdot \frac{\Delta T_{measured\;FEM}}{\Delta T_{strips\;FEM}}$$
